# Study on the spillover effects of tail risks in the supply chain of China’s pharmaceutical industry

**DOI:** 10.3389/fpubh.2026.1704501

**Published:** 2026-02-04

**Authors:** Cheng Wang, Mengnan Xu, Mengyue Xu

**Affiliations:** 1 School of Pharmaceutical Economics and Management, Anhui University of Chinese Medicine, Hefei, China; 2 Key Laboratory of Data Science & Innovative Development of Traditional Chinese Medicine, Philosophy and Social Sciences of Anhui Province, Hefei, China

**Keywords:** pharmaceutical industry supply chain, public health event, risk spillover, tail risk, TENET

## Abstract

**Introduction:**

In the context of heightened economic uncertainty and frequent extreme events, enhancing the resilience of pharmaceutical supply chains, safeguarding their security and stability, and promoting high-quality development in China’s pharmaceutical industry have become pressing issues requiring in-depth research.

**Methods:**

This study takes China’s pharmaceutical industry from January 1, 2012, to March 31, 2023, as the research subject. The TENET method is employed to construct a tail risk network for the pharmaceutical supply chain. We examine its structural characteristics and dynamic temporal patterns, while analyzing variations in risk spillover effects across different tail risk events. Results: At the overall supply chain level, tail risks exhibit notable time-varying characteristics, with total connectedness rising significantly during risk events. At the module level, the production module serves as the primary source of both risk input and output. Cross-module analysis reveals clustering characteristics in risk spillovers between the production and distribution modules. Additionally, bidirectional spillovers are observed between the service and distribution modules, as well as between these modules and the production module. At the institutional level, the in-degree and out-degree of pharmaceutical institutions are not correlated with market capitalization. Hengrui Pharmaceuticals, Aier Eye Hospital, and Fosun Pharma are identified as systemically important institutions in the supply chain. Furthermore, the characteristics of the risk network vary under different tail risk events: financial crises elevate the overall risk level of the supply chain, whereas public health events do not significantly impact the overall risk level. Nonetheless, tail events universally increase the frequency of risk propagation within the supply chain.

**Discussion:**

While the TENET model employed in this study serves as a powerful tool for analyzing tail risk networks, it possesses inherent limitations. Future research could integrate structured econometric models, such as the introduction of exogenous instrumental variables, or adopt high-frequency data causal discovery techniques. These approaches would help disentangle intrinsic causal pathways and further reveal the “topology of risk transmission.

## 1 Introduction

The safety of the pharmaceutical industry supply chain is a crucial component of national economic security and public health security. In recent years, escalating U.S.-China trade tensions, the outbreak of the COVID-19 pandemic, and the frequent occurrence of local wars and other unexpected events have triggered extreme risks that not only caused severe fluctuations in financial markets but also exerted significant shocks on supply chains and value chains across various industries (1). For example, constrained by U.S. multilateral export controls, leading enterprises in China’s biopharmaceutical sector are facing a “supply chain disruption” crisis. Frequent pressures from pharmaceutical policies and unexpected black swan events in the industry have led to a significant reshuffling of pharmaceutical companies and a comprehensive restructuring of supply chains. The global pandemic has also caused multiple instances of widespread disruptions to the pharmaceutical supply chain. Therefore, in the current context of considerable economic uncertainty and the frequent occurrence of extreme events, enhancing the pharmaceutical supply chain’s risk resilience, ensuring its security and stability, and thereby promoting the high-quality development of China’s pharmaceutical industry supply chain, has become a pressing issue requiring urgent attention and in-depth research.

The supply chain is composed of two types of elements: entities and structures (2). Its characteristics of systemicity, interconnectedness, and complexity determine that supply chain risks are inherently contagious. Current research on supply chain risk contagion largely focuses on the enterprise and industry levels. For instance, studies have explored how internal corporate crises trigger a “resonance effect” through the supply chain (3–6) or examined the impact of sudden external events on supply chain vulnerability (7–9). However, there is limited research at the industry level investigating the spillover effects of supply chain risks.

Tail risk often involves systemic risk and chain reactions, making the study of tail risk an important aspect of preventing systemic risk. The premise of tail risk research is the accurate identification and measurement of risk levels. Classic methods for measuring tail risk include Value at Risk (VaR) and Expected Shortfall (ES). Considering the contagious nature of risks, single risk measurement theories can no longer suffice. As a result, Adrian and Brunnermeier (10) proposed Conditional Value at Risk (CoVaR) based on VaR to study the risk contribution of a single institution to another institution or the system during risk events, namely, the level of risk spillover. In order to better characterize the networked interconnections of risks between institutions, a series of systemic risk measurement methods based on network correlation have been proposed, such as marginal expected loss (11), generalized variance decomposition (12), Copula models and others. The network correlation model has been widely applied in the financial field (13–16), and it is currently one of the more mature methods for studying tail risk spillovers. However, most network correlation models still assume that the overall model is linear, neglecting the nonlinear characteristics between institutions, which may lead to biased results (17–19).

Härdle et al. (20) developed the Tail Event driven NETwork (TENET) model based on a Single Index Quantile Regression (SIM) model, which effectively addresses the nonlinear issues in network correlation. The TENET model has been widely applied in financial markets and related fields (21–26). Research results indicate that the TENET model is more efficient in risk identification under tail risk conditions. It not only captures the nonlinear characteristics of tail risks but also effectively captures their spillover effects. This capability makes it a promising tool for analyzing complex industrial ecosystems.

However, existing research predominantly focuses on risk spillovers across countries, financial markets, or broad economic sectors, typically treating aggregate markets or entire industries as network nodes (15, 17). While valuable for macro-level systemic risk assessment, such an approach fails to uncover the complex, module-based risk transmission mechanisms within the supply chain of a specific industry. Concurrently, systematic investigation into tail risks within the pharmaceutical supply chain remains notably scarce. The limited extant literature on pharmaceutical supply chain risks largely relies on linear analytical paradigms—such as Granger causality tests, Vector Autoregression models, or Copula methods focusing on upstream-downstream linkages (7–9). These studies, often grounded in traditional supply chain risk theory with an emphasis on structural mapping and operational flows, tend to overlook the intense nonlinear feedback effects and the dynamic, time-varying nature of risk contagion among different functional modules within the supply chain network.

Bridging this gap, our study innovatively applies the TENET framework to the context of a complex industrial supply chain. We take the Chinese pharmaceutical industry from January 1, 2012, to March 31, 2023, as the research object and use the TENET method to construct a tail risk network for the pharmaceutical industry supply chain. This network specifically characterizes the tail risk correlations and their dynamic time-varying features among the three major functional modules derived from supply chain theory: pharmaceutical production, distribution, and healthcare services.

Our research has the following three contributions: First, methodological innovation. We extend the application of the TENET model from the domain of macro-financial risk analysis to the context of meso-level industrial supply chain risk analysis. By constructing a three-module (production, distribution, and service) tail risk network for the pharmaceutical supply chain, it transcends the limitations of traditional approaches that treat an industry as a homogeneous entity or focus solely on bilateral relationships (23, 24). This enables a more granular characterization of the heterogeneous transmission dynamics of risks across different functional modules within the supply chain. Second, empirical discovery innovation. Existing TENET research seldom systematically compares the differential impacts of different types of extreme events on the same risk network (22, 26). We not only reveal the static network topology but also, through a dynamic comparison of two distinct tail shocks—the financial crisis and the global public health event—provide an in-depth analysis of how they differentially reshape the risk transmission pathways within the pharmaceutical supply chain. This finding offers new empirical evidence for understanding the transmission mechanisms of different shock sources. Third, integration of research perspectives. We organically integrate modularity theory from supply chain management with tail risk network theory from financial econometrics, providing an operational analytical framework for interdisciplinary research on industrial resilience.

The rest of this paper proceeds as follows. Section 2 constructs the TENET mode. Section 3 presents the sample and variables. Section 4 presents the empirical results. Section 5 research on the tail risk contagion network of supply chains under different risk event shocks. Section 6 conclusions and recommendations.

## 2 TENET model construction

The TENET model is completed in three steps: The first step involves estimating the CoVaR values of individual institutions (Equations 1–5). The second step entails constructing a tail risk contagion matrix and conducting network connectivity analysis (Equations 6–14). The third step focuses on identifying institutions of systemic importance (Equations 15–16).

### 2.1 CoVaR estimation

First, we estimate the VaR of the weekly returns of individual institutions in the supply chain. The formula is set as follows:


(1) $$P(X_{i,t}\leq \mathrm{Va}R_{i,t,\tau })=\tau$$

(2) $$X_{i,t}=\alpha_{i}+\gamma_{i}M_{t-1}+\varepsilon_{i,t}$$

(3) $${\hat{\mathrm{VaR}}}_{i,t,\tau }=\hat{\alpha_{i}}+\hat{\gamma_{i}}M_{t-1}$$

where $X_{\mathrm{it}}$ represents the logarithmic return of listed institution i at time t, τ represents the quantile level, and $M_{t-1}$ represents the set of macro state variables affecting tail risk.

Then, we establish a single-index model (SIM) to estimate CoVaR, which is used to measure the tail risk spillover effects between supply chain institutions. The formula is set as follows:


(4) $$X_{j,t}=g(\beta_{j|R_{j}}^{T}R_{j,t})+\varepsilon_{j,t}$$

(5) $${\hat{\text{CoVaR}}\tilde{R}}_{j,t,\tau }^{\text{TENET}}=\hat{g}({\hat{\beta }}_{j|{\tilde{R}}_{j}}{\tilde{R}}_{j,t})$$

where g(⋅) is a smooth link function, representing the nonlinear interactive influence of other institutions in the supply chain on institution j. $R_{j,t}=\{X_{-j},M_{t-1},B_{j,t-1}\}$, where $X_{-j}$ represents the set of logarithmic returns of institutions other than institution j, $M_{t-1}$ is the set of macro state variables, $B_{j,t-1}$ is the set of characteristic variables, and $\beta_{j|R_{j}}={\left\{\beta_{j|X_{-j}},\beta_{j|M_{t-1}},\beta_{j|B_{j,t-1}}\right\}}^{T}$ is the vector set of regression coefficients corresponding to the variables. To avoid overfitting the model, the MACE double iteration method is used to estimate g(⋅) and $\beta_{j|R_{j}}$ (27, 28). ${\hat{\text{CoVaR}}\tilde{R}}_{j,t,\tau }^{\text{TENET}}$ represents the conditional Value at Risk based on TENET, ${\hat{\mathrm{VaR}}}_{-j,t,\tau }=\{{\hat{\mathrm{VaR}}}_{1,t,\tau },{\hat{\mathrm{VaR}}}_{2,t,\tau },\cdots ,{\hat{\mathrm{VaR}}}_{j-1,t,\tau },{\hat{\mathrm{VaR}}}_{j+1,t,\tau },\cdots ,{\hat{\mathrm{VaR}}}_{k,t,\tau }\}$ represents the VaR values corresponding to the other k−1 institutions excluding institution j.

### 2.2 Construction of the tail risk contagion matrix

To construct the tail risk network, it is necessary to identify nodes (institutions) and edges (risk spillovers). The following formula can be used to measure the marginal impact of VaR risk from other institutions on the CoVaR risk of an institution:


(6) $${\hat{d}}_{j|{\hat{R}}_{j}}=\frac{\partial \hat{g}({\hat{\beta }}_{j|{\tilde{R}}_{j}}{\tilde{R}}_{j,t})}{\partial {\tilde{R}}_{j,t}}={\hat{g}}^{'}({\hat{\beta }}_{j|{\tilde{R}}_{j}}{\tilde{R}}_{j,t}){\hat{\beta }}_{j|{\tilde{R}}_{j}}$$

where ${\hat{d}}_{j|{\hat{R}}_{j}}={\left\{{\hat{d}}_{j|\mathrm{Va}R_{-j,t}},{\hat{d}}_{j|M_{t-1}},{\hat{d}}_{j|B_{j,t-1}}\right\}}^{T}$, represents the gradient vector of the marginal effects of covariates estimated when $R_{j,t}={\tilde{R}}_{j,t}$. ${\hat{d}}_{j|\mathrm{Va}R_{-j,t}}$ can measure the risk spillover effects between supply chain institutions and can be used to depict the dynamic evolution of institutions in a network. Since our primary focus is on the tail risk spillover effects between institutions, we only consider the partial derivatives ${\hat{d}}_{j|\mathrm{Va}R_{i,t}}$ of institution j relative to other institutions i=1,2,⋯,j−1,j+1,⋯,kwith i≠j (hereinafter abbreviated as ${\hat{d}}_{j|i}$), excluding the partial derivatives of financial environment variables and industry characteristic variables. Taking the ω window as an example, the following k×k order adjacency matrix containing the risk spillover effects ${\hat{d}}_{j|i}$ between institutions is constructed:


(7) $$T_{\omega }=(\begin{array}{ccccc} 0 & |{{\hat{d}}^{\omega }}_{1|2}| & |{{\hat{d}}^{\omega }}_{1|3}| & \cdots & |{{\hat{d}}^{\omega }}_{1|k}|\\ |{{\hat{d}}^{\omega }}_{2|1}| & 0 & |{{\hat{d}}^{\omega }}_{2|3}| & \cdots & |{{\hat{d}}^{\omega }}_{2|k}|\\ |{{\hat{d}}^{\omega }}_{3|1}| & |{{\hat{d}}^{\omega }}_{3|2}| & 0 & \cdots & |{{\hat{d}}^{\omega }}_{3|k}|\\ \vdots & \vdots & \vdots & \ddots & \vdots \\ |{{\hat{d}}^{\omega }}_{k|1}| & |{{\hat{d}}^{\omega }}_{k|2}| & |{{\hat{d}}^{\omega }}_{k|3}| & \cdots & 0 \end{array})$$

where $|{\hat{d}}_{j|i}^{\omega }|$ is the absolute value of ${\hat{d}}_{j|i}^{\omega }$, representing the tail risk spillover level from institution i to institution j.

Based on the tail risk contagion matrix, four categories of indicators can be constructed to examine the connectivity of the tail risk network: Total Connectedness (TC), Module Connectedness (MC), Module Cross Connectedness (MCC), and Institution Connectedness (IC). The specific formulas can be expressed as follows:


(8) $$TC_{\omega }=\sum_{j=1}^{K}\sum_{i=1}^{k}|{\hat{d}}_{i|j}^{w}|=\sum_{j=1}^{K}\sum_{i=1}^{k}|{\hat{d}}_{j|i}^{w}|$$

where $TC_{\omega }$ refers to the overall network connectivity of the supply chain under the ω window, which is derived by summing up the tail risk spillover levels of each node in the supply chain.


(9) $$MC_{g,\omega }^{\text{in}}=\sum_{j\in g}\sum_{i=1}^{k}|{\hat{d}}_{j|i}^{w}|$$

(10) $$MC_{g,\omega }^{\text{out}}=\sum_{j\in g}\sum_{i=1}^{k}|{\hat{d}}_{i|j}^{w}|$$

where MC represents the tail risk effects of the three major modules under the ω window, g=1,2,3 corresponds to the production, distribution, and service modules, respectively. $MC_{1,\omega }^{\text{in}}$ denotes the sum of the tail risk spillover effects received by the production module from all other modules, referred to as the in-degree. $MC_{1,\omega }^{\text{out}}$ represents the sum of the tail risk spillover effects from the production module to all other modules, referred to as the out-degree.


(11) $$\mathrm{MC}C_{g_{y}|g_{x},\omega }^{\text{in}}=\frac{1}{k_{g_{y}}k_{g_{x}}}\sum_{j\in g_{y}}\sum_{i\in g_{x}}|{\hat{d}}_{j|i}^{w}|$$

(12) $$\mathrm{MC}C_{g_{y}|g_{x},\omega }^{\text{out}}=\frac{1}{k_{g_{y}}k_{g_{x}}}\sum_{j\in g_{y}}\sum_{i\in g_{x}}|{\hat{d}}_{i|j}^{w}|$$

where MCC represents the tail risk effects between pairwise modules of the supply chain at the ω time window, $k_{g_{x}}$ and $k_{g_{y}}$ denote the number of institutions in the $g_{x}$ and $g_{y}$ supply chain modules, respectively. If studying the tail risk effect of a specific institution within $g_{x}$ on the remaining institutions, then $k_{g_{x}}=k_{g_{y}}-1,g_{x}=g_{y},j\neq i$. Similarly, it is divided into in-degree ($\mathrm{MC}{C_{g_{y}}}_{|g_{x},\omega }^{\text{in}}$) and out-degree ($\mathrm{MC}{C_{g_{y}}}_{|g_{x},\omega }^{\text{out}}$) categories.


(13) $$IC_{p,\omega }^{\text{in}}=\sum_{i=1}^{k}|{\hat{d}}_{j|i}^{w}|$$

(14) $$IC_{p,\omega }^{\text{out}}=\sum_{j=1}^{k}|{\hat{d}}_{i|j}^{w}|$$

where IC represents the sum of the tail risk spillover effects from a specific institution to all other institutions in the supply chain under the ω window, which is divided into in-degree ($IC_{p,\omega }^{\text{in}}$) and out-degree ($IC_{p,\omega }^{\text{out}}$) categories.

### 2.3 Identification of systemically important institutions

The impact of tail risk events on the supply chain primarily affects the entire supply chain through key links or core institutions. Therefore, it is necessary to identify systemically important institutions in the context of supply chain tail risk. We refer to Härdle et al. (20) and introduces the Systemic Risk Receiver Index (SRRI) and the Systemic Risk Emitter Index (SREI). By considering both the connectivity and scale of institutions, the two core risk management concepts of “too big to fail” and “too connected to fail” are combined to identify systemically important institutions. The SRRI and SREI indices for institution j at window w are defined as:


(15) $$\mathrm{SRR}I_{j,\omega }=\mathrm{AM}C_{j,\omega }\sum_{i\in k_{\omega }^{\text{in}}}(|{\hat{d}}_{j|i}^{\omega }|\mathrm{AM}C_{i,\omega })$$

(16) $$\mathrm{SRE}I_{j,\omega }=\mathrm{AM}C_{j,\omega }\sum_{i\in k_{\omega }^{\text{out}}}(|{\hat{d}}_{i|j}^{\omega }|\mathrm{AM}C_{i,\omega })$$

where $\mathrm{AM}C_{j,w}$ represents the market value of institution j at window w, indicating the scale of the institution.

### 2.4 Model implementation details

The empirical analysis of our study is conducted in the R 4.3.2 environment. The core estimation of the TENET model utilized the quantreg package for quantile regression. To estimate the nonparametric link function g(⋅) in the single-index model, we implemented the Maximum Aggregated Curvature Estimation(MACE) algorithm proposed by Härdle et al. (20). This algorithm optimizes parameters through a double-iteration process, with the convergence criterion set as follows: iteration stops when the norm of relative changes in all coefficient estimates is less than 10–5 between two consecutive iterations, ensuring estimation stability. All network analysis and visualizations were performed using the igraph and ggplot2 packages.

## 3 Sample and variables

### 3.1 Sample selection

Based on the 2021 revised version of the Shenyin Wanguo industry classification and the business structure of medical institutions, we divide the supply chain of the pharmaceutical industry into three major modules: pharmaceutical production, pharmaceutical distribution, and medical services (excluding medical devices). The selected sample period is from January 1, 2012, to March 31, 2023, which includes financial events such as the “stock market crash” and public health events like the COVID-19 pandemic, facilitating the study of tail risk spillover effects in the pharmaceutical industry supply chain. To enhance the stability and reliability of the data and to ensure the validity of the model estimation, the sample selection adheres to the following criteria: First, institutions must have been established before 2008. This requirement ensures that, at the beginning of our sample period (2012), the firms had achieved a stable operational status and possessed a sufficiently long financial history, thereby enhancing the reliability of their risk profile estimation. Second, institutions without complete trading records or those suspended for more than one year during the sample period are excluded to meet the TENET model’s requirement for continuous time-series data and to guarantee the effectiveness of the model estimation. Ultimately, 57 institutions (40 pharmaceutical production enterprises, 12 pharmaceutical distribution enterprises, and 5 medical service institutions) are selected as the research subjects.

### 3.2 Variable design

Table 1 presents the descriptive statistics of the weekly returns of the selected 57 institutions. The data is sourced from the Wind database. The weekly return is defined as $X_{j,t}=\ln(CP_{j,t}/CP_{j,t-1})$, where $CP_{j,t}$ is the closing price of institution j in week t. The selection of macro state variables $M_{t-1}$ primarily follows the approach of Härdle et al. (20), including market volatility, stock market returns, term spread, short-term liquidity spread, credit spread, and weekly returns of the real estate industry. The macro state variables are selected from the Wind database. The selection of industry characteristic variables mainly including leverage ratio, firm size, maturity mismatch ratio, and market-to-book ratio. The industry characteristic variables are derived from the quarterly balance sheets in the CSMAR database. We use the cubic spline interpolation method to convert the frequency of industry characteristic data into weekly data. Specific definitions are provided in Table 2.

**Table 1:** Descriptive statistics of weekly returns of 57 listed institutions.

Supply chain module	Institution name	ID	Stock code	Mean	Standard deviation	Median	Minimum	Maximum
Pharmaceutical production	Fengyuan Pharmaceutical	X1	000153	0.001	0.051	0.000	−0.341	0.328
	Dong-E–E-Jiao	X2	000423	0.001	0.039	0.002	−0.166	0.181
	Livzon Pharmaceutical	X3	000513	0.003	0.044	0.003	−0.236	0.178
	Yunnan Baiyao	X4	000538	0.002	0.036	0.000	−0.129	0.201
	Hainan Haiyao	X5	000566	0.000	0.052	0.000	−0.261	0.238
	Northeast Pharmaceutical	X6	000597	0.001	0.051	0.004	−0.344	0.179
	Jilin Aodong Pharmaceutical	X7	000623	0.001	0.039	0.002	−0.227	0.165
	Apeloa Pharmaceutical	X8	000739	0.003	0.049	0.003	−0.238	0.174
	Xinhua Pharmaceutical	X9	000756	0.003	0.060	0.001	−0.305	0.410
	Tonghua Jinma Pharmaceutical Dongbao	X10	000766	0.000	0.049	0.000	−0.255	0.236
	PKU HealthCare	X11	000788	0.000	0.054	0.000	−0.290	0.412
	Jinling Pharmaceutical	X12	000919	0.000	0.046	0.003	−0.225	0.235
	Guangji Pharmaceutical	X13	000952	0.001	0.049	0.001	−0.349	0.191
	Jiuzhitang	X14	000989	0.001	0.051	0.002	−0.242	0.435
	Jingxin Pharmaceutical	X15	002020	0.003	0.044	0.001	−0.184	0.264
	Hisun Pharmaceutical	X16	002099	0.001	0.054	0.000	−0.325	0.281
	China Resources Double-Crane	X17	600062	0.001	0.040	0.000	−0.227	0.147
	Humanwell Healthcare	X18	600079	0.002	0.046	0.003	−0.253	0.274
	Tongrentang	X19	600085	0.003	0.044	0.001	−0.216	0.181
	Fosun Pharmaceutical	X20	600196	0.004	0.049	0.004	−0.231	0.237
	Jiangsu Wuzhong	X21	600200	0.000	0.061	0.000	−0.365	0.353
	Tibet Pharmaceutical	X22	600211	0.003	0.058	0.000	−0.331	0.368
	Zhejiang Medicine	X23	600216	0.001	0.048	0.002	−0.233	0.209
	Talong Pharmaceutical	X24	600222	0.001	0.052	0.002	−0.363	0.311
	Zhongheng Group	X25	600252	0.000	0.045	0.001	−0.279	0.200
	Hengrui Pharmaceuticals	X26	600276	0.005	0.036	0.004	−0.141	0.151
	Lingrui Pharmaceutical	X27	600285	0.002	0.044	0.004	−0.233	0.267
	Livzon Pharmaceutical	X28	600380	0.002	0.055	0.003	−0.289	0.315
	Sinopharm Modern	X29	600420	0.003	0.050	0.001	−0.368	0.259
	Tianjin Pharmaceutical	X30	600488	0.001	0.043	0.002	−0.300	0.195
	Tasly Pharmaceutical	X31	600535	0.002	0.041	0.001	−0.197	0.176
	Kanion Pharmaceutical	X32	600557	0.001	0.043	−0.001	−0.178	0.185
	Jichuan Pharmaceutical	X33	600566	0.003	0.046	0.002	−0.333	0.296
	Conba Pharmaceutical	X34	600572	0.001	0.040	0.000	−0.247	0.204
	Yibai Pharmaceutical	X35	600594	0.000	0.046	0.001	−0.325	0.165
	Jiangzhong Pharmaceutical	X36	600750	−0.001	0.043	0.000	−0.192	0.242
	Hanshang Group	X37	600774	0.003	0.053	0.004	−0.377	0.240
	North China Pharmaceutical	X38	600812	0.000	0.057	0.002	−0.345	0.378
	Haixin Group	X39	600851	0.001	0.056	0.003	−0.354	0.243
	Mayinglong	X40	600993	0.001	0.050	0.003	−0.265	0.249
Pharmaceutical distribution	Sinopharm Group	X41	000028	0.001	0.040	0.002	−0.202	0.143
	Neptunus Bioengineering	X42	000078	0.001	0.060	0.000	−0.370	0.364
	Intco Medical	X43	000411	0.000	0.060	0.000	−0.370	0.426
	Zhejiang Zhenyuan	X44	000705	0.002	0.058	0.003	−0.384	0.431
	Jiashitang	X45	002462	0.001	0.051	0.000	−0.348	0.292
	China Meheco	X46	600056	0.001	0.051	0.000	−0.347	0.362
	Kaikai Industrial	X47	600272	0.000	0.056	0.004	−0.371	0.270
	Sinopharm A-Share	X48	600511	0.000	0.048	0.000	−0.314	0.227
	Nanjing Pharmaceutical	X49	600713	0.001	0.046	0.002	−0.331	0.180
	Renmin Tongtai	X50	600829	0.000	0.052	0.000	−0.379	0.236
	First Medicine	X51	600833	0.000	0.056	0.001	−0.382	0.238
	Shanghai Pharmaceutical	X52	601607	0.002	0.042	0.003	−0.249	0.189
Medical services	International Medical	X53	000516	0.003	0.055	0.000	−0.281	0.279
	Meinian Health	X54	002044	0.002	0.062	0.000	−0.390	0.426
	Bright Eye Hospital	X55	002524	0.001	0.064	0.000	−0.379	0.270
	Aier Eye Hospital	X56	300015	0.005	0.045	0.006	−0.210	0.187
	Topchoice Medical	X57	600763	0.005	0.051	0.005	−0.209	0.196

**Table 2:** Industry characteristics variables and macroeconomic state variables.

Category	Variable name	Definition	Frequency
Industry characteristic variables	Leverage ratio	Total assets/Total equity	Quarterly
	Firm size	Logarithm of total assets	Quarterly
	Maturity mismatch ratio	(Short-term debt−Monetary funds)/Total liabilities	Quarterly
	Price-to-book ratio	Price-to-book ratio	Quarterly
Macroeconomic state variables	Market volatility	Conditional variance of the SSE composite index return series estimated by GARCH(1,1) model	Weekly
	Stock market return	Weekly return series of the SSE composite index	Weekly
	Liquidity spread	Difference between the 3-month interbank lending rate and the 3-month treasury bond yield	Weekly
	Term spread	Difference between the 10-year treasury bond yield and the 6-month treasury bond yield	Weekly
	Credit spread	Difference between the 10-year treasury bond yield and the 10-year AAA corporate bond yield	Weekly
	Real estate industry return	Weekly return of the CSI real estate index	Weekly

## 4 Empirical results

### 4.1 Analysis of the overall tail risk spillover effect in the supply chain

**Figure 1:**
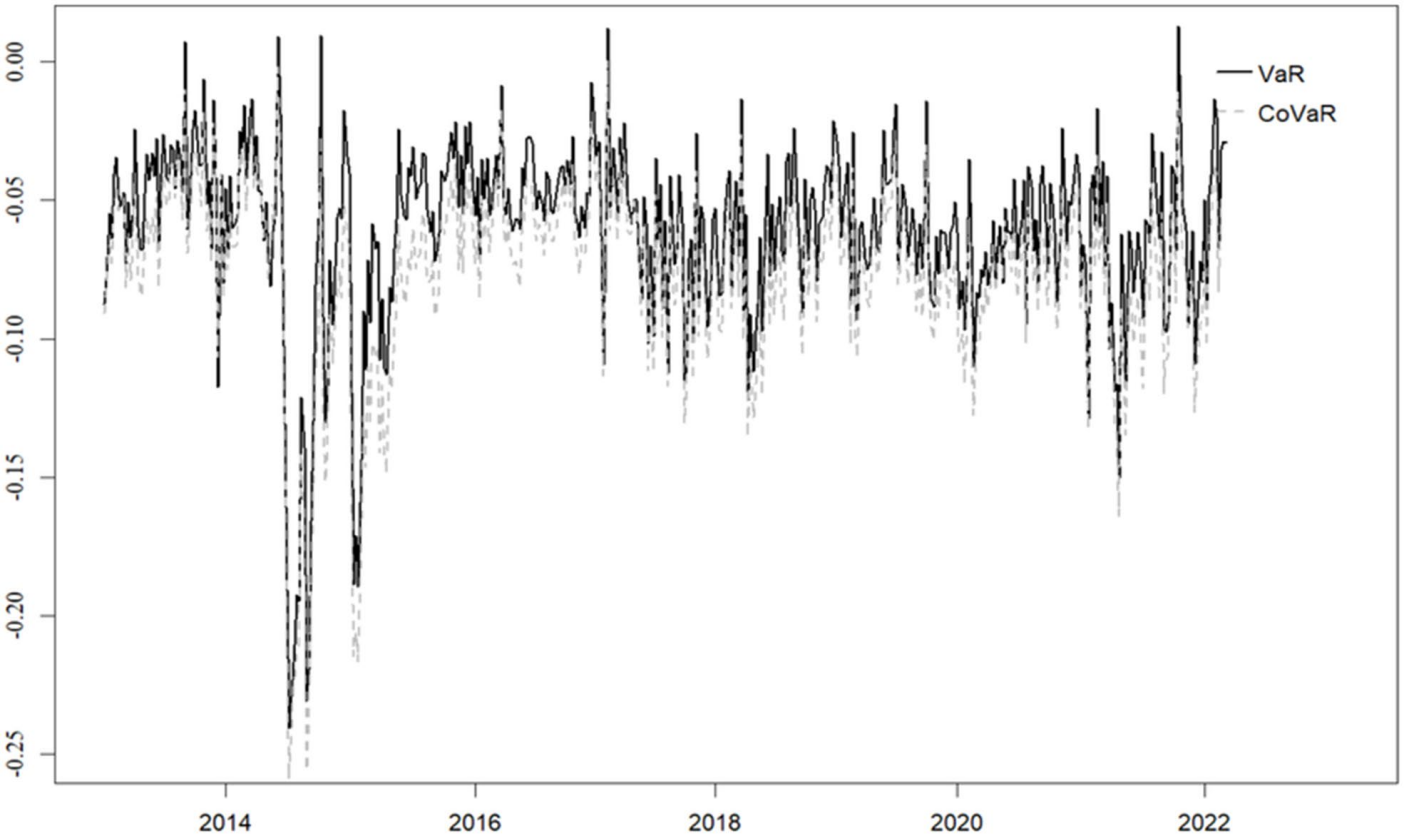
The VaR, CoVaR values of pharmaceutical organisations.

**Figure 2:**
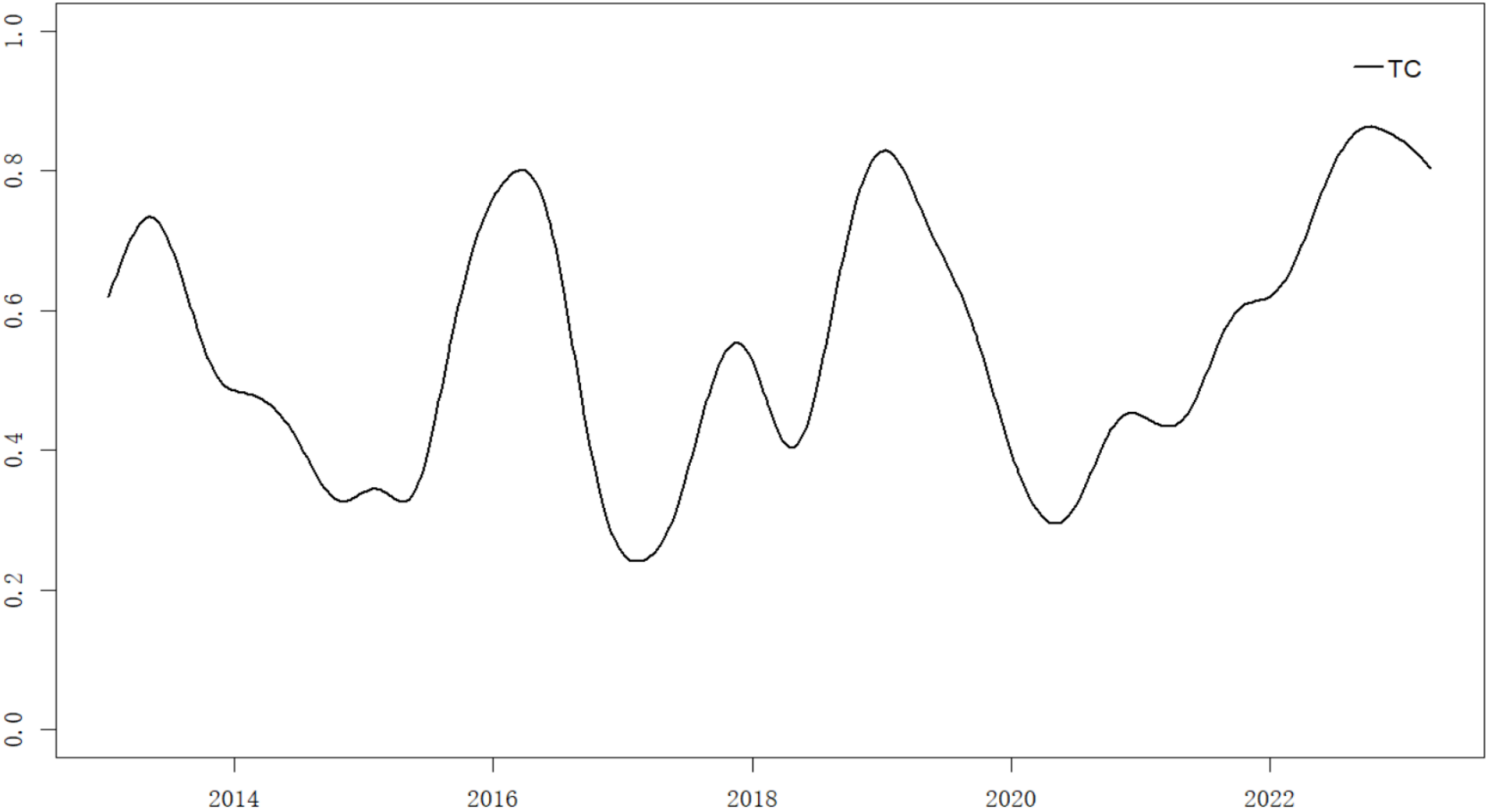
TC of supply chain risk spillover networks in the pharmaceutical industry.

We set the quantile level to τ=0.01, and the rolling window size to ω=51, with the entire period being 575. After calculating using Equations 1–6, the VaR and ${\hat{\text{CoVaR}}}^{\text{TENET}}$ values for pharmaceutical institutions and the overall supply chain correlation level $TC_{\omega }$ were obtained. The specific results are shown in Figures 1, 2.

The VaR and ${\hat{\text{CoVaR}}}^{\text{TENET}}$ values in Figure 1 are derived from the average risk indicators of 57 pharmaceutical institutions. As can be seen from Figure 1, the fluctuation trends of VaR and CoVaR values for pharmaceutical institutions are quite similar. That is, when the VaR value of pharmaceutical institutions is higher, the corresponding risk spillover effect is also stronger. The most significant changes occurred around 2015, indicating that the value at risk and risk spillover effects of pharmaceutical institutions are greater during this period. However, VaR and CoVaR values can only reflect part of the market conditions in the pharmaceutical industry, and further analysis of other risk indicators in the pharmaceutical industry is needed.

Figure 2 illustrates the fluctuation trend of total connectedness within pharmaceutical institutions during the sample period. The analysis reveals two key findings: First, the total connectedness demonstrates significant time-varying characteristics, particularly showing elevated levels during tail events. Second, four pronounced volatility episodes emerge during the observation window: (1) The first peak around 2013 correlates with the “Money Crunch” event. This financial strain triggers simultaneous declines across capital market sectors. The pharmaceutical stock market experiences substantial impacts as rising bank interest rates increase corporate financing costs and strain cash flows, triggering a resonance effect among institutions in the pharmaceutical supply chain. (2) The second peak in 2016 aligns with China’s stock market crash and circuit breaker mechanism implementation. These systemic financial shocks produce extensive market repercussions, evidenced by a 30.34% decline in the Shenwan Medicine and Biotechnology Industry Index within one-month post-crash. Two subsequent circuit breaker events in January 2016 further depress the index by 15.83%. (3) The third peak around 2019 corresponds to the COVID-19 outbreak. While China’s stringent containment measures effectively curb epidemic spread, they simultaneously induce economic stagnation. The healthcare system faces severe supply–demand imbalances for medical resources, causing rapid risk escalation across pharmaceutical supply chain nodes. (4) The fourth peak emerges in late 2022 following China’s pandemic control deregulation. The prolonged pandemic (2020–2022) depresses economic growth and industry demand. Particularly in December 2022, sudden lifting of restrictions creates acute supply chain risks for “four-category medicines” (antiviral, antipyretic, cough, and cold remedies), dramatically increasing pharmaceutical supply chain vulnerabilities.

### 4.2 Analysis of tail risk spillover effects among supply chain modules

**Figure 3:**
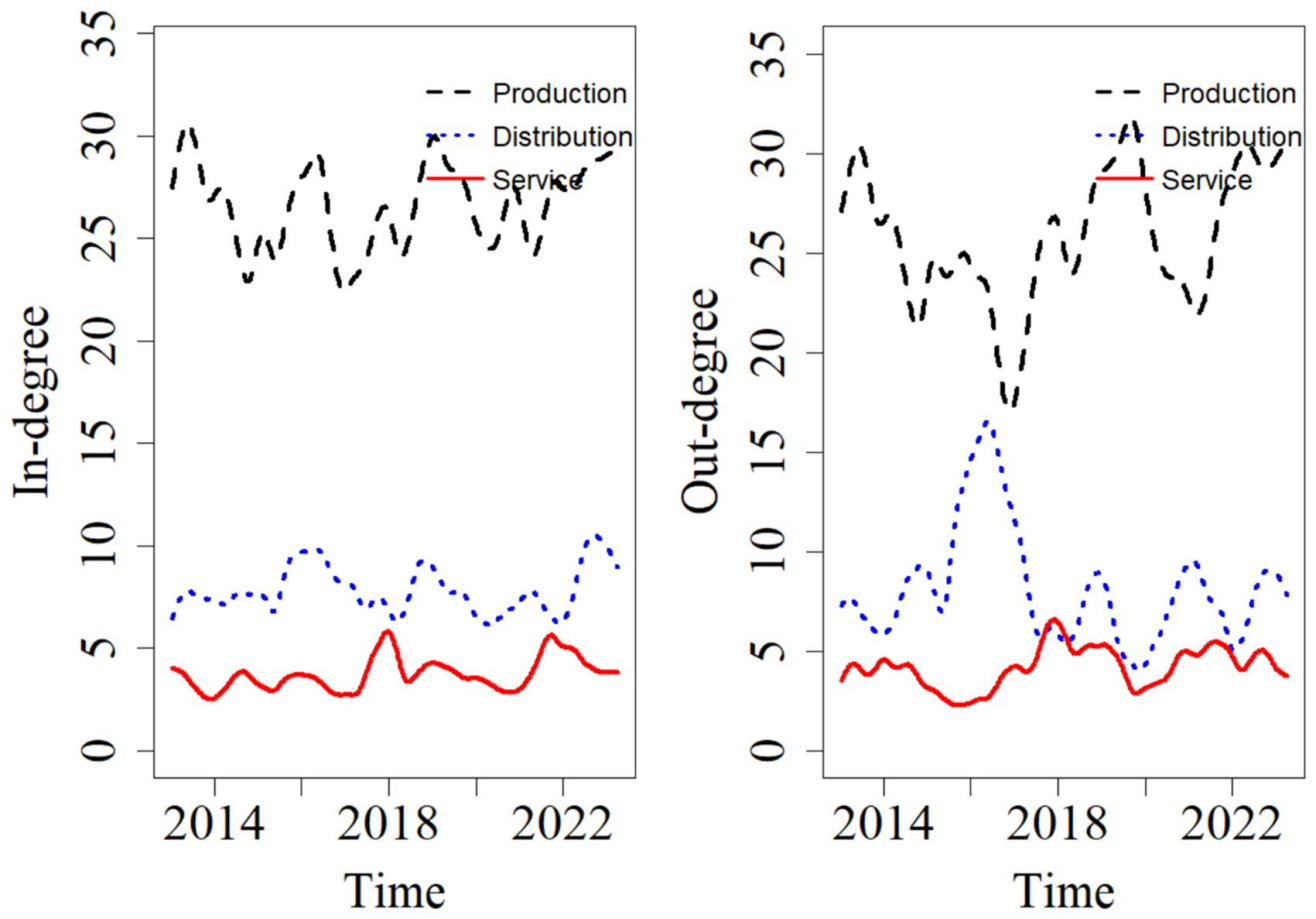
In-degree and out-degree of supply chain modules in the pharmaceutical industry.

Figure 3 shows the trends in in-degree and out-degree changes for the three major modules of the pharmaceutical industry supply chain: production, distribution, and service. As seen in Figure 3, the production module has the highest in-degree and out-degree, accounting for 69.7 and 67.6% of the input and output of tail risk spillover effects in the pharmaceutical supply chain, respectively. This indicates that the production module is a critical component of the pharmaceutical supply chain, serving as a sensitive node in the risk transmission process. It is susceptible to influences from the distribution and service modules, while also significantly affecting the efficiency of pharmaceutical distribution and the effectiveness of medical services. The reasons for this are as follows: pharmaceutical production includes multiple stages, such as drug research and development, manufacturing, and quality control, involving a wide range of fields and numerous risk points. Additionally, the production process is characterized by high complexity, significant control challenges, unstable raw material sources, and stringent regulatory standards, all of which contribute to substantial potential risks. Therefore, risk management in the pharmaceutical industry should place greater emphasis on controlling risks within the production module to prevent chain-wide risks originating from the production stage.

It is noteworthy that in 2016, the risk spillover from the distribution module increases significantly, with its risk spillover level approaching that of the production module. The reason for this is as follows: in March 2016, influenced by the Shandong vaccine incident, a nationwide campaign to regulate pharmaceutical distribution gains momentum. In April of the same year, the State Council decides to promote the “two-invoice system.” The implementation of this system effectively reduces the number of steps in the drug distribution process, improves drug supply efficiency, and ensures medication safety. However, it also poses significant challenges to distribution companies, such as the expansion of accounts receivable, the decline of transfer business, and the pressure to build a comprehensive distribution network, leading to short-term chaos in the pharmaceutical distribution sector. On the other hand, the two-invoice system is beneficial for production companies and medical service institutions, as it standardizes the distribution process and reduces operational risks. Therefore, during this period, the risk spillover level of the production module decreases. Before the policy was implemented, the distribution chain exhibited a multi-layered structure, with multiple intermediaries between manufacturers and healthcare institutions. While this structure dispersed financial and operational risks across multiple entities, it also created opaque and elongated payment chains and inventory buffers that could absorb certain external shocks to some extent. After the policy was implemented, the distribution chain was significantly shortened, effectively reducing the number of transaction links and intermediaries. Although this enhanced transparency and lowered drug prices, it also concentrated transactional and credit risks onto a smaller number of large-scale distributors and transferred greater liquidity pressure upstream to manufacturers. Consequently, the financial linkages between distributors and manufacturers became more direct and tighter. The financial health of distributors (e.g., cash flow constraints, credit defaults) began to transmit more rapidly and significantly to manufacturers, whose accounts receivable and order stability became more sensitive to distributor performance. Conversely, disruptions on the production side (e.g., quality incidents, supply delays) also propagated more directly to distributors, whose inventory flexibility declined due to the streamlined chain. This explains why, after 2016, we not only observed an increase in overall connectedness between the two modules but also found a significant enhancement in bidirectional and symmetric spillover intensity, indicating that risk coupling had become more pronounced.

### 4.3 Analysis of cross-module tail risk spillover effects

Table 3 summarizes the top 10 pharmaceutical companies with the highest risk inputs and outputs among the top three entities in each module. The entities in the production module include Hengui pharma, Fuliying pharma, Yunnan Baiyao, Tongfang pharma, and Huanfang pharma. The entities in the distribution module include Shanghai pharma, China pharma, and Gupuyihe. The entities in the Service Module include Aihe ophthalmology and Tongzhi medical. Through analysis, it is found that: (1) The risk transmission within the production module exhibits a tendency to cluster. Five parent pharmaceutical companies collectively have 10 input institutions, with 8 belonging to the production module, and 1 each from the distribution and service modules. Similarly, these five companies collectively have 13 output institutions, with 11 in the production module, and 1 each in the distribution and service modules. This is due to the fact that pharmaceutical products, as special goods, require strict adherence to quality management systems, process standards, and regulatory requirements during production, which inherently carries a high-risk level. Additionally, there are often close collaborative relationships among pharmaceutical manufacturing enterprises, such as consignment manufacturing and joint R&D efforts. These tight interactions and collaborations lead to frequent exchanges of information, materials, and personnel, thereby increasing the likelihood of risk transmission. Consequently, the manufacturing stage is more prone to generating incidents involving risks that can infect each other. (2) The production module and the distribution module as well as the service module exhibit a bidirectional risk spillage characteristic. 2 service institutions have a total of 6 connected input institutions, with 4 from the production module, 1 each from the distribution and service modules; 2 service institutions have a total of 6 connected output institutions, with 3 from the production module and 3 from the service module. Three distribution institutions have 9 connected input institutions in total, with 4 from the production module and 5 from the distribution module; 3 distribution institutions have 7 connected input institutions in total, with 3 from the production module and 4 from the distribution module. This is because the production module serves as an upstream for both the distribution module and the service module, with its product quality, supply speed, and reliability directly impacting the operational efficiency of the distribution chain and the service level at the service stage. Therefore, risk incidents in the production stage will propagate through the supply chain to the distribution stage and the service stage. Additionally, China has stringent regulations and pharmaceutical management policies for the medical industry, with risks arising from both the distribution and healthcare stages leading to industry-wide and large-scale restructuring of the supply chain. Consequently, there is a bidirectional risk spillage between the production module and the service module as well as the distribution module.

**Table 3:** Top 3 organisations with the largest risk inputs and largest risk outputs in the top 10 organisations by market capitalisation.

Market cap ranking	Institution name	Risk input entities	Risk output entities
1	Hengrui Pharmaceuticals	Aier Eye Hospital, North China Pharmaceutical, Tibet Pharmaceutical	North China Pharmaceutical, Yunnan Baiyao, Aier Eye Hospital
2	Aier Eye Hospital	Topchoice Medical, Tibet Pharmaceutical, First Pharmaceutical	Hengrui Pharmaceuticals, Fosun Pharmaceutical, Yunnan Baiyao
3	Shanghai Pharmaceuticals	Nanjing Pharmaceutical, China Pharmaceutical, Huarun Double Crane	Sinopharm Group, China Pharmaceutical, Huarun Double Crane
4	Fosun Pharmaceutical	Livzon Pharmaceutical, China Pharmaceutical, Tibet Pharmaceutical	Tibet Pharmaceutical, Livzon Pharmaceutical, China Pharmaceutical
5	Yunnan Baiyao	Fosun Pharmaceutical, Aier Eye Hospital, Dong-E–E-Jiao	Dong-E–E-Jiao, Tongrentang, Hengrui Pharmaceuticals
6	Tongrentang	Yunnan Baiyao, Mayinglong, Fosun Pharmaceutical	Jilin Aodong, Dong-E–E-Jiao, Guangji Pharmaceutical
7	Humanwell Pharmaceutical	Mayinglong, North China Pharmaceutical, Zhongheng Group	Mayinglong, Dong-E–E-Jiao, Zhongheng Group
8	Topchoice Medical	Jiuzhitang, Haisco Pharmaceutical, Mayinglong	Aier Eye Hospital, International Medical, Bright Eye Hospital
9	China Pharmaceutical	Fosun Pharmaceutical, Northeast Pharmaceutical, Jiashitang	Sinopharm Group, Fosun Pharmaceutical, Shanghai Pharmaceuticals
10	Sinopharm Group	Sinopharm Group, Shanghai Pharmaceuticals, Sinopharm Modern	Sinopharm Group, Tonghua Jinma, Fosun Pharmaceutical

### 4.4 Analysis of tail risk spillover effects in medical institutions

Table 4 summarizes the in-degree and out-degree of individual medical institutions, with the top 10 ranked due to space limitations. The following conclusions can be drawn from the table: (1) The in-degree and out-degree of individual medical institutions do not depend on their market size. For example, Jiangsu Wuzhong, Bright Eye Hospital, and First Pharmaceutical. Although these institutions are small in scale, they play a significant role in the supply chain and have high levels of risk input and output. Regulatory authorities should cautiously address these small and medium-sized institutions that are active in terms of in-degree and out-degree to prevent potential systemic risks, ensuring the regulatory principle of “too connected to fail.” (2) High vigilance should be maintained regarding the risks of medical service institutions and pharmaceutical distribution enterprises, with strengthened regulatory measures. Notably, among the top 10 individual medical institutions in terms of in-degree and out-degree, pharmaceutical production institutions account for 5 (12.5% of the total sample within the module), distribution institutions account for 3 (25% of the total sample within the module), and medical service institutions account for 2 (40% of the total sample within the module). This indicates that medical service institutions and pharmaceutical distribution enterprises have high individual risk coefficients. Although the overall in-degree and out-degree of the distribution and medical service modules are low, there are still institutions within these modules with high-risk coefficients.

**Table 4:** Ranking of individual pharmaceutical organizations in terms of inward and outward movement.

Top 10 institutions by in-degree		Top 10 institutions by out-degree			
Institution name	In-degree	Market cap ranking	Institution name	Out-degree	Market cap ranking
Jiangsu Wuzhong	660.880	44	Bright Eye Hospital	472.748	52
Neptune Bio	636.652	11	Jiangsu Wuzhong	464.483	44
Haixin Shares	587.713	46	Sinopharm Group	438.993	14
Tailong Pharmaceutical	582.853	50	International Medical	432.078	18
Kaikai Industrial	581.774	57	Tibet Pharmaceutical	430.451	35
Peking University Pharmaceutical	526.742	49	Neptune Bio	419.359	11
Bright Eye Hospital	502.852	52	Northeast Pharmaceutical	414.628	25
North China Pharmaceutical	495.348	20	Tonghua Jinma	413.986	45
First Pharmaceutical	480.094	55	Xinhua Pharmaceutical	406.859	26
Topchoice Medical	467.809	8	First Pharmaceutical	405.330	55

### 4.5 Identification of systemically important institutions in the supply chain

In the analysis of Table 4, the size of the medical institutions themselves is not considered, which may affect the actual impact of risk spillover. By comprehensively considering the size and influence of the medical institutions, the systemically important institutions in the supply chain are identified. Table 5 lists the top 10 institutions ranked by SRRI (Supply Chain Risk Receiver Index) and SREI (Supply Chain Risk Emitter Index) values, along with their corresponding market capitalization rankings. From Table 5, the following conclusions are drawn: (1) Institutions with larger market capitalizations are more likely to be both risk receivers and risk emitters in the supply chain. Whether ranked by SRRI or SREI, institutions with larger market capitalizations are positioned higher. Therefore, regulatory authorities remain vigilant about these larger institutions potentially triggering systemic risks and ensure their safety and stability. (2) Hengrui Pharmaceuticals, Aier Eye Hospital, and Fosun Pharmaceutical are identified as systemically important institutions in the supply chain. These three institutions are ranked within the top 3 in both SRRI and SREI rankings. Regulatory authorities strengthen risk identification and monitoring for these three institutions. Notably, we identify systemically important institutions based on their centrality within the financial risk network, following the logic of being ‘too big to fail’ or ‘too connected to fail.’ This definition focuses on their potential impact on the financial stability of the entire industry. We acknowledge that this may not fully overlap with the definition of ‘critical institutions’ from a public health perspective, which emphasizes the provision of irreplaceable life-saving drugs or the guarantee of grassroots supply. However, understanding the vulnerabilities of these financial hubs is crucial. If such institutions encounter financial distress, the resulting pressure could transmit to numerous small and medium-sized enterprises upstream and downstream—including those critical from a public health standpoint—through channels such as supply chain credit, equity investments, and business expectations. This, in turn, could trigger or exacerbate broader medication supply risks.

**Table 5:** Ranking of total out and total in supply chain risks.

Top 10 institutions by SRRI			Top 10 institutions by SREI		
Institution name	SRRI (*10^23^)	Market cap ranking	Institution name	SREI (*10^23^)	Market cap ranking
Hengrui Pharmaceuticals	1.331	1	Aier Eye Hospital	1.004	2
Aier Eye Hospital	0.927	2	Fosun Pharmaceutical	0.844	4
Fosun Pharmaceutical	0.667	4	Hengrui Pharmaceuticals	0.778	1
Yunnan Baiyao	0.474	5	Topchoice Medical	0.412	8
Shanghai Pharmaceuticals	0.428	3	Yunnan BaiyaoYunnan Baiyao	0.377	5
Tongrentang	0.263	6	Shanghai Pharmaceuticals	0.325	3
China Pharmaceutical	0.207	9	Tongrentang	0.218	6
Livzon Pharmaceutical	0.184	13	China Pharmaceutical	0.203	9
Humanwell Pharmaceutical	0.155	7	Livzon Pharmaceutical	0.187	13
Sinopharm Group	0.148	10	Humanwell Pharmaceutical	0.164	7

In the table, (*10^23^) indicates that the corresponding coefficient is multiplied by 10 to the power of 23.

## 5 Research on the tail risk contagion network of supply chains under different risk event shocks

We further characterize the tail risk spillover network features of the pharmaceutical industry supply chain under shocks from different types of risk events, exploring the paths and patterns of tail risk contagion. Therefore, the tail risk spillover networks of the pharmaceutical industry supply chain are depicted for three different periods: the full sample period (2012.01–2023.03), the financial crisis event period (2014.07–2016.06), and the public health event period (2019.01–2020.12).

### 5.1 Full sample period

**Figure 4:**
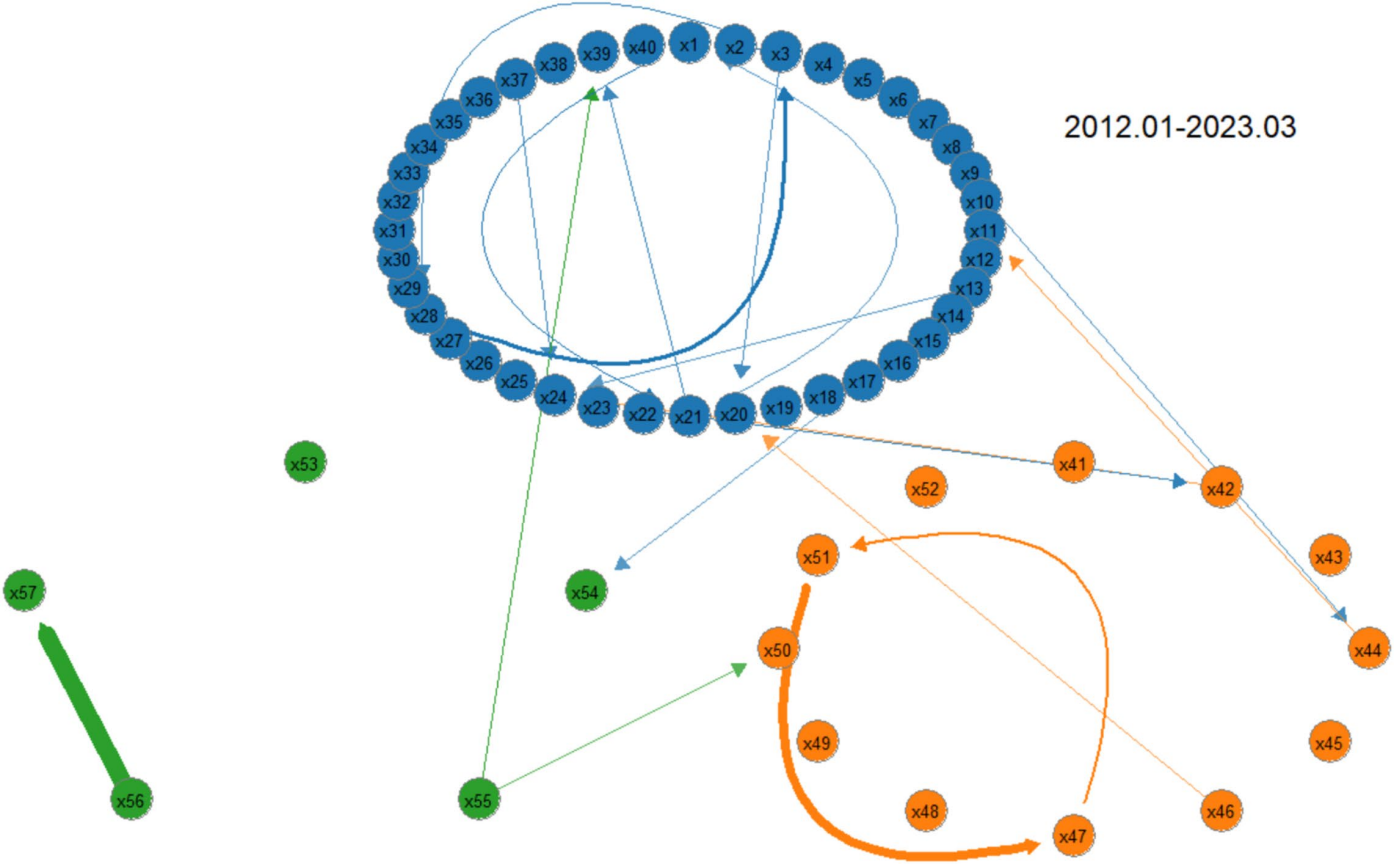
Supply chain tail risk spillover network in the pharmaceutical industry over the full sample period. The above diagram shows the production module, with the lower left being the medical service module and the lower right being the distribution module. Below this, further details will be provided without repeating the same information at length.

To better present the risk propagation characteristics between sectors, a threshold is set to make the main connections clearer. The thickness of the edges in the graph represents the magnitude of the risk spillover effect, with thicker edges indicating stronger spillover effects, and the direction of the arrows represents the input and output of risk. From Figure 4, the following observations can be made: (1) Risk propagation among institutions exhibits clustering, meaning risks are more likely to spread and diffuse within the same module. (2) There is cross-module tail risk spillover in the supply chain, with the production module being the main source of risk and the most frequent point of risk exchange. (3) It is necessary to be vigilant about the high-risk spillover from Aier Eye Hospital to Topchoice Medical, as well as the bidirectional risk spillover between First Pharmaceutical and Kaikai Industrial, to prevent “tail events.” These findings further validate the conclusions drawn earlier in the study.

### 5.2 Financial crisis event impact

**Figure 5:**
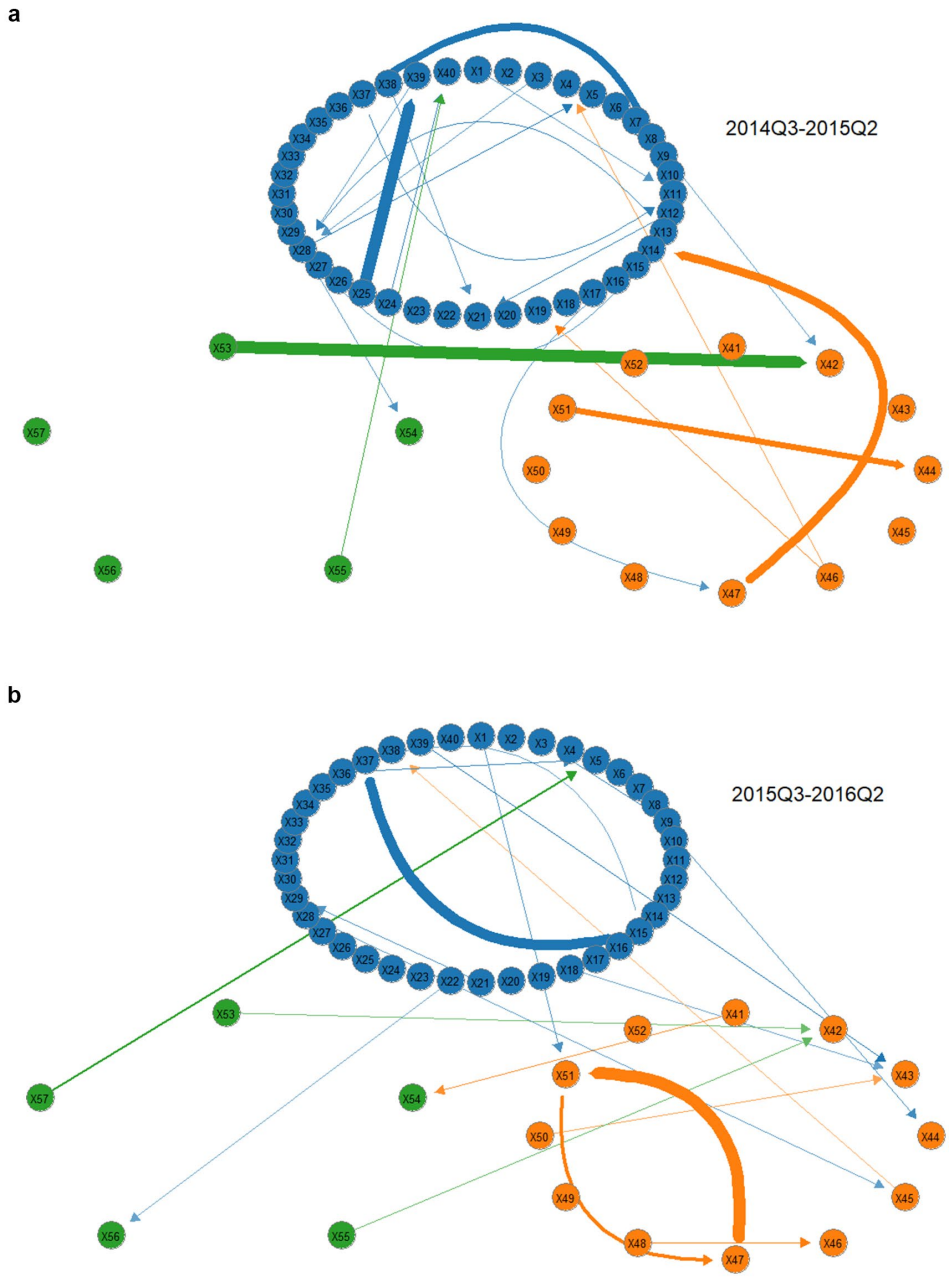
**(a)** Tail risk spillover network before the onset of the financial crisis. **(b)** Network of tail risk spillovers after the onset of the financial crisis.

Figures 5a,b, along with Table 6, illustrate the tail risk network of the pharmaceutical industry supply chain during the financial crisis from 2014Q3 to 2016Q2. Key findings include: (1) The financial crisis increases the overall risk level of the pharmaceutical industry supply chain. The overall risk level of the pharmaceutical supply chain rose after the crisis, increasing from 32.682 before the crisis to 42.971 during the crisis. (2) The production module remains the primary source of risk. Risk interactions within the production module and cross-module risk spillovers are frequent both before and after the crisis. (3) The distribution module is most impacted by the financial crisis. After the crisis, risk transmission activities increased significantly, with the number of associated edges rising from 7 to 13 during the crisis. Additionally, internal risk propagation within the distribution sector also became more pronounced. This aligns with the industry and the promotion of the “Two Tickets System” implemented in 2016.

**Table 6:** Changes in network connectivity before and after the onset of the financial crisis.

Connectedness	2014Q3–2015Q2			2015Q3–2016Q2		
	Production	Distribution	Service	Production	Distribution	Service
In-degree	22.258	7.254	3.171	28.857	10.888	3.226
Out-degree	24.399	5.590	2.694	22.859	17.387	2.725
Total connectedness	32.682			42.971		

### 5.3 Impact of public health events

**Figure 6:**
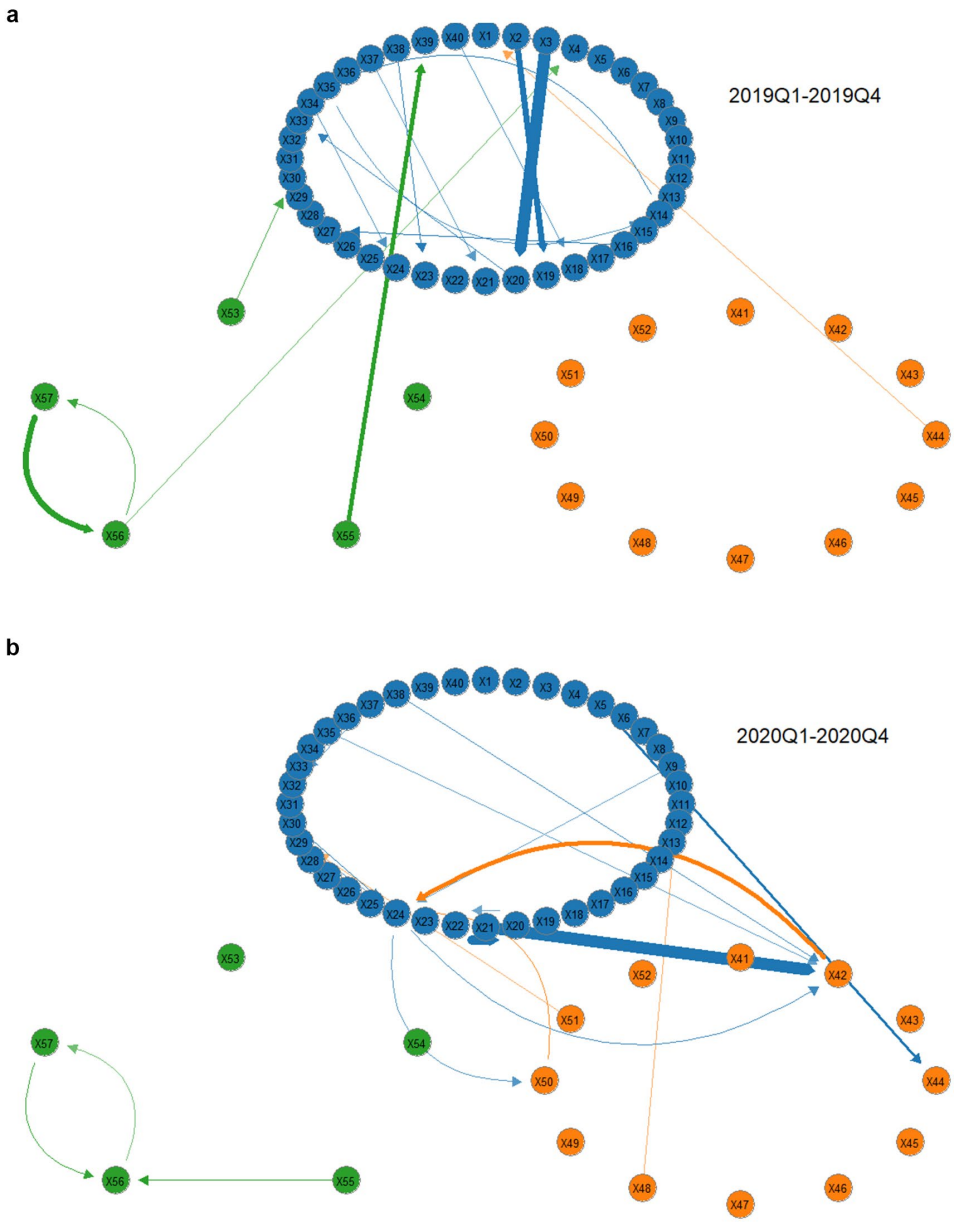
**(a)** COVID-19 front-tail risk spillover network. **(b)** COVID-19 post-tail risk spillover network.

Figures 6a,b and Table 7 illustrate the tail risk network of the pharmaceutical industry supply chain during the public health event period from 2019Q1 to 2020Q4. The key findings are as follows: (1) After the outbreak of COVID-19, the overall risk level of the pharmaceutical industry supply chain increases slightly, but the rise remains minimal. This is mainly because, at the early stage of the pandemic, the Chinese government efficiently activates a first-level emergency response mechanism, makes scientific decisions, deploys resources rationally, and implements precise prevention and control measures, keeping potential risks within a reasonable range. (2) The pharmaceutical distribution module serves as the main source of tail risk spillover in the supply chain. While the risk input level of the pharmaceutical distribution module does not change significantly, its risk output level increases sharply. The bidirectional risk spillover between the distribution and production modules becomes more pronounced after the outbreak. This happens because a series of government policies, such as special funding projects, tax incentives, and streamlined approval processes, reduce the risk output of pharmaceutical production institutions. However, the pandemic significantly increases pharmaceutical distribution costs, amplifies existing issues in warehouse resource integration and logistics distribution networks, and raises risk levels in the distribution sector. Consequently, the risk spillover to the medical services sector also rises.

**Table 7:** Changes in network connectivity before and after COVID-19.

Connectedness	2019Q1–2019Q4			2020Q1–2020Q4		
	Production	Distribution	Service	Production	Distribution	Service
In-degree	25.557	7.277	4.282	27.691	7.574	3.473
Out-degree	29.434	3.783	3.899	23.287	9.873	5.579
Total connectedness	37.116			38.739		

### 5.4 Robustness tests

To ensure the reliability of the research conclusions, we conducted sensitivity analyses. Specifically, we adjusted the rolling window to 102 weeks (*ω* = 102) and the quantile level to 5% (*τ* = 0.05), then re-ran the TENET model. The results show that: (1) the fluctuation pattern of the overall supply chain risk connectivity (TC) remains largely consistent with that of the baseline model; (2) the production module consistently maintains its dominant role in risk transmission under all settings; (3) the identification of systemically important institutions is robust, with Hengrui Pharmaceuticals, Aier Eye Hospital, and Fosun Pharma consistently ranking at the top; and (4) Financial crises lead to an increase in the overall risk level of supply chains, but public health events do not result in significant growth in supply chain risk exposure. This indicates that the main findings of this study are not sensitive to parameter choices.

#### 5.4.1 Systemic tail risk and spillover effects

**Figure 7:**
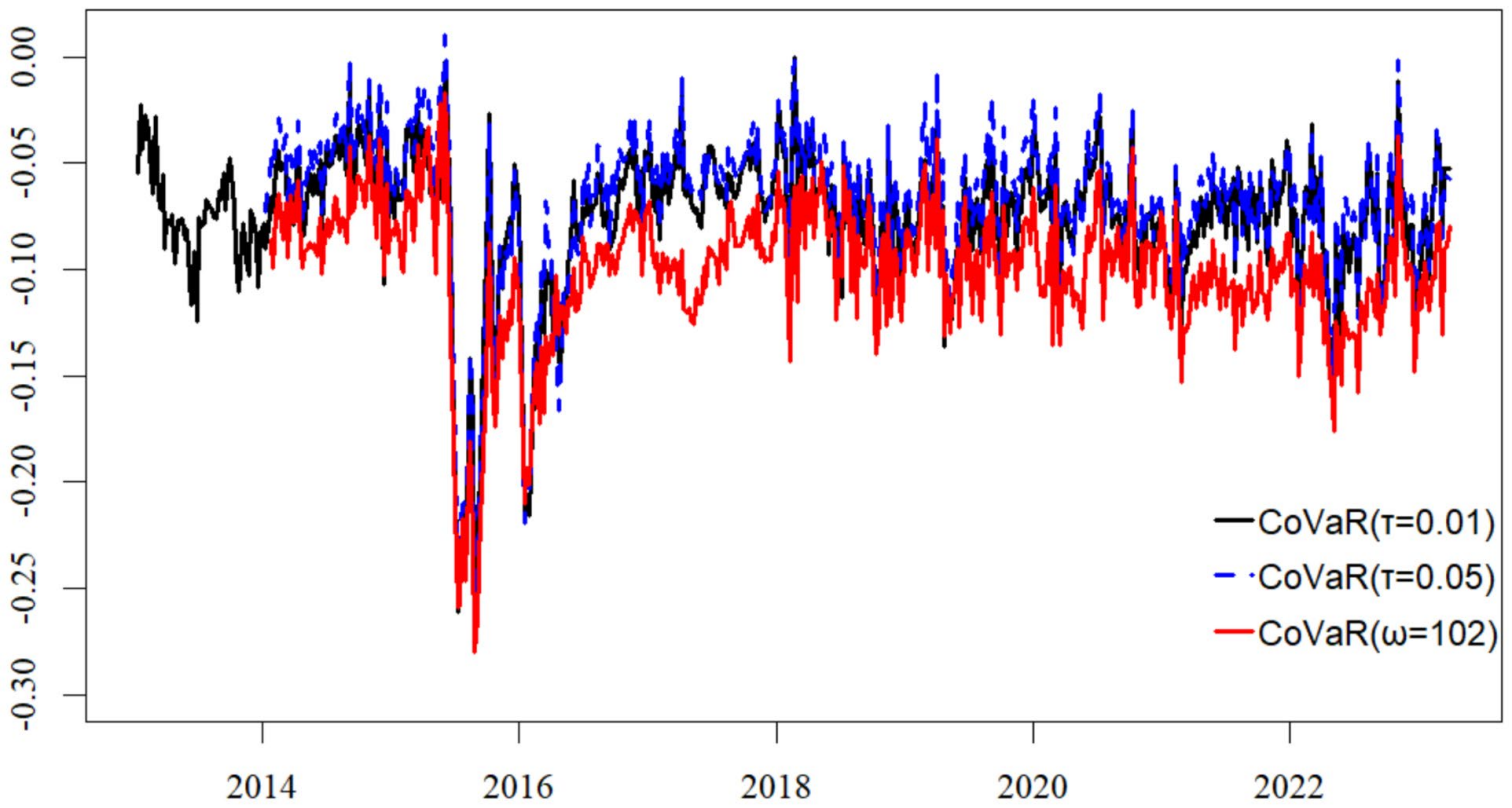
CoVaR estimates across different quantile levels and rolling windows.

**Figure 8:**
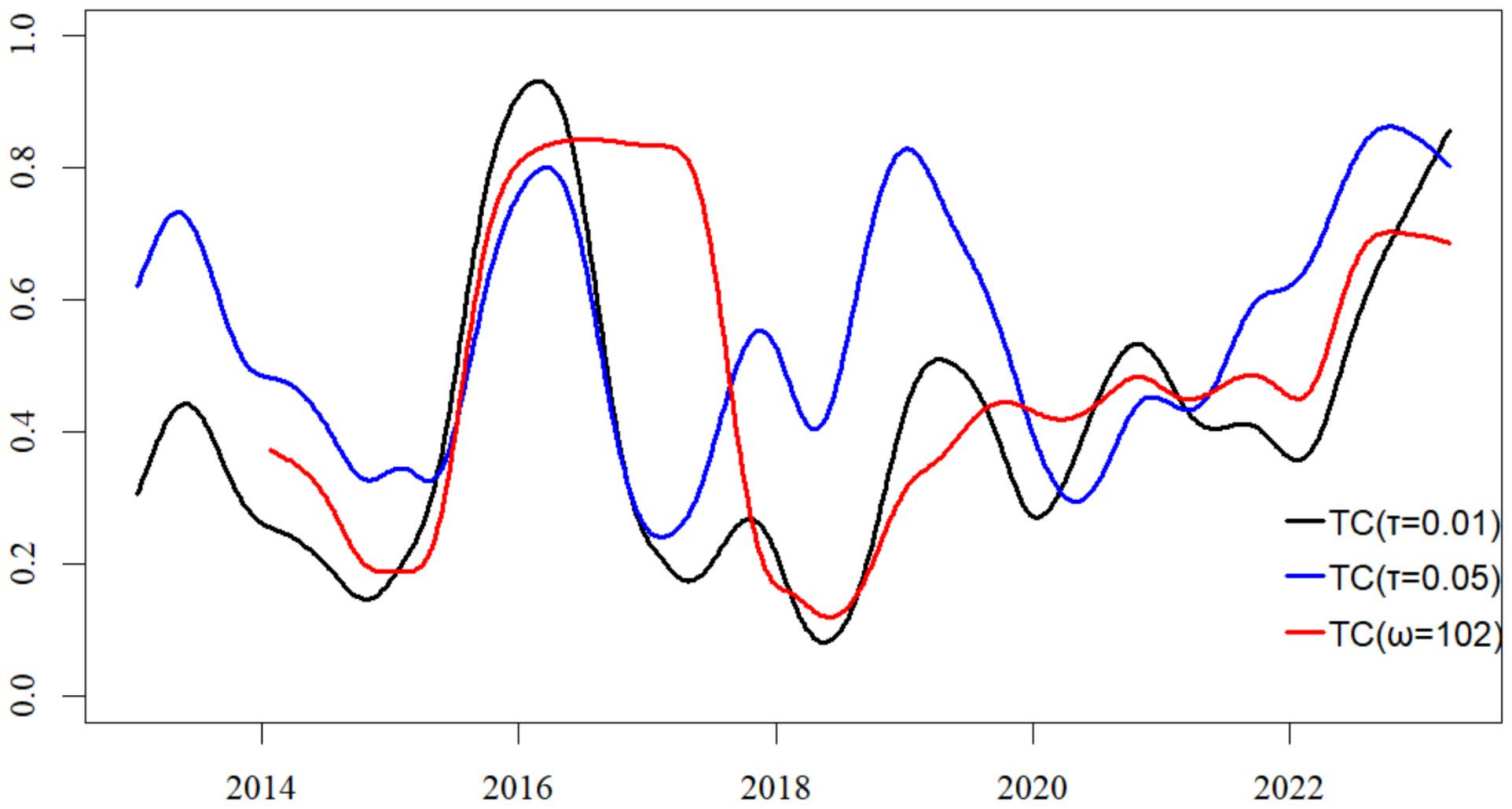
Total connectedness of risk spillover networks under varying quantile levels and rolling windows.

Figures 7, 8 demonstrate that the trajectories of Total Connectedness (TC) and CoVaR remain highly consistent in both their trends and magnitudes across the baseline specification (τ = 0.01) and the robustness check with adjusted parameters (τ = 0.05, ω = 102). This consistency indicates that the core time-varying dynamics of supply chain systemic risk are not sensitive to the chosen quantile level or the length of the rolling estimation window.

In our robustness test with adjusted quantile level and rolling window length (τ = 0.05, ω = 102), we find that the production module consistently maintains its systemically critical role in the risk network. Our analysis shows that both its in-degree and out-degree measures remain significantly higher than those observed in the distribution and service modules, demonstrating that its dominance in risk reception and transmission is robust to parameter specifications. These results reinforce our preliminary conclusion that the production module functions not only as the primary recipient and propagator of supply chain risk spillovers but also constitutes the central source of structural vulnerability within the pharmaceutical supply chain network. We therefore conclude that this dual role remains consistently evident across multiple modeling frameworks.

Our analysis reveals that extending the rolling window length produces the expected smoothing effect, substantially reducing short-term fluctuations in the estimated results. This finding further suggests that when employing such time-varying models for forward-looking risk assessment or early warning, a critical trade-off must be made between the depth of historical information and the timeliness of real-time data.

#### 5.4.2 Identification of systemically important institutions

**Figure 9:**
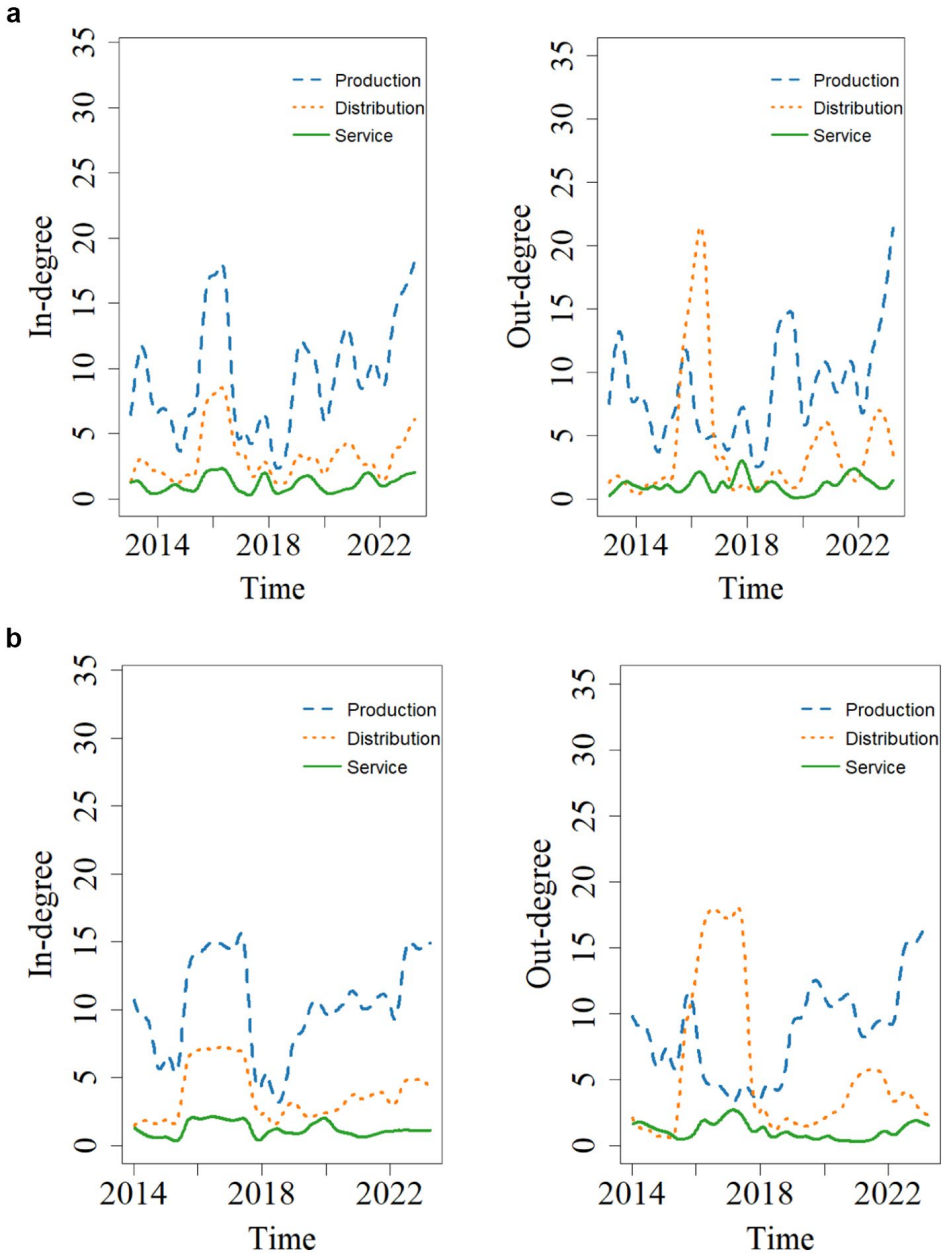
**(a)** The in-degree and out-degree of each supply chain module (*τ* = 0.05). **(b)** The in-degree and out-degree of each supply chain module (*ω* = 102).

The findings from our robustness tests (τ = 0.05 and ω = 102), as shown in Figures 9a,b, corroborate the stable and significant relationship between market capitalization and systemic importance. Notably, Hengrui Pharmaceuticals, Aier Eye Hospital, and Fosun Pharmaceutical persistently appear at the forefront of both the Systemic Risk Receiver Index (SRRI) and the Systemic Risk Emitter Index (SREI) rankings. This consistent pattern validates the strong robustness of their identification as systemically important institutions within the supply chain, indicating that their individual risk dynamics possess a structurally significant impact on the stability of the entire pharmaceutical sector (see Tables 8, 9).

**Table 8:** Ranking of total out and total in supply chain risks (τ = 0.05).

Top 10 institutions by SRRI			Top 10 institutions by SREI		
Institution name	SRRI (*10^22^)	Market cap ranking	Institution name	SREI (*10^22^)	Market cap ranking
Hengrui Pharmaceuticals	3.210	1	Fosun Pharmaceutical	4.160	4
Aier Eye Hospital	2.650	2	Topchoice Medical	2.160	11
Fosun Pharmaceutical	1.800	4	Neptune Bio-Engineering	1.790	8
Shanghai Pharmaceuticals Holding	1.740	3	Aier Eye Hospital	1.330	9
Yunnan Baiyao Group	0.938	5	China Meheco	1.140	13
China Meheco	0.845	9	Hengrui Pharmaceuticals	1.070	20
Tongrentang	0.783	6	Livzon Pharmaceutical	1.050	1
Livzon Pharmaceutical	0.741	13	Tongrentang	1.010	6
North China Pharmaceutical	0.701	20	North China Pharmaceutical	0.774	2
Joincare Pharmaceutical	0.657	12	Humanwell Pharmaceutical	0.497	21

In the table, (*10^22^) indicates that the corresponding coefficient is multiplied by 10 to the power of 22.

**Table 9:** Ranking of total out and total in supply chain risks (ω = 102).

Top 10 institutions by SRRI			Top 10 institutions by SREI		
Institution name	SRRI (*10^22^)	Market cap ranking	Institution name	SREI (*10^22^)	Market cap ranking
Aier Eye Hospital	3.510	2	Fosun Pharmaceutical	4.580	4
Hengrui Pharmaceuticals	3.350	1	Topchoice Medical	2.460	8
Fosun Pharmaceutical	1.570	4	Neptune Bio-Engineering	2.000	11
Shanghai Pharmaceuticals	1.400	3	Aier Eye Hospital	1.100	2
Yunnan Baiyao	1.140	5	China Meheco	1.020	9
China Meheco	0.867	9	Hengrui Pharmaceuticals	0.936	1
China National Medicines	0.776	14	Livzon Pharmaceutical	0.838	13
Tongrentang	0.770	6	Tongrentang	0.757	6
North China Pharmaceutical	0.712	20	North China Pharmaceutical	0.738	20
Livzon Pharmaceutical	0.426	13	Humanwell Pharmaceutical	0.496	7

In the table, (*10^22^) indicates that the corresponding coefficient is multiplied by 10 to the power of 22.

#### 5.4.3 Heterogeneous effects of risk events

As demonstrated in Tables 10–13, while adjusting the quantile level (τ) and rolling window length (ω) alters the absolute magnitude of the risk measures, the core qualitative conclusions remain robust: financial crises significantly amplify the overall risk level of the pharmaceutical supply chain, the production module consistently acts as the systemic risk hub during crises, and the distribution module is the most affected. This consistency confirms that our findings are not statistical artifacts of a specific model configuration but reflect intrinsic and robust structural characteristics as well as dynamic patterns within the pharmaceutical supply chain risk network. Consequently, policy recommendations derived from these conclusions possess greater reliability and practical relevance.

**Table 10:** Effects of financial crises(τ = 0.05).

Connectedness	2014Q3–2015Q2			2015Q3–2016Q2		
	Production	Distribution	Service	Production	Distribution	Service
In-degree	14.193	4.015	2.040	41.984	19.574	5.346
Out-degree	13.979	3.932	2.338	21.669	41.702	3.532
Total connectedness	20.248			66.903		

**Table 11:** Effects of financial *n*(ω = 10^2^).

Connectedness	2014Q3–2015Q2			2015Q3–2016Q2		
	Production	Distribution	Service	Production	Distribution	Service
In-degree	16.360	4.436	1.283	34.677	17.017	4.793
Out-degree	17.285	2.184	2.609	20.837	32.724	2.926
Total connectedness	22.079			56.487		

**Table 12:** Effects of health crises (τ = 0.05).

Connectedness	2019Q1–2019Q4			2020Q1–2020Q4		
	Production	Distribution	Service	Production	Distribution	Service
In-degree	26.417	7.479	3.546	26.872	9.208	1.524
Out-degree	32.807	3.584	1.051	23.328	12.422	1.854
Total connectedness	37.442			37.604		

**Table 13:** Effects of health crises (ω = 10^2^).

Connectedness	2019Q1–2019Q4			2020Q1–2020Q4		
	Production	Distribution	Service	Production	Distribution	Service
In-degree	23.955	5.907	3.498	27.058	7.892	2.836
Out-degree	27.854	4.056	1.450	28.286	8.384	1.116
Total connectedness	33.360			37.786		

## 6 Conclusions and recommendations

We study the tail risk spillover effects in the pharmaceutical industry supply chain during the period from January 1, 2012, to March 31, 2023. The main content includes: using the TENET model to construct a tail risk network of China’s pharmaceutical industry supply chain to capture the tail risk spillover effects at different supply chain nodes; applying network analysis theory to characterize the tail risk association network and its dynamic time-varying characteristics in the three main modules of the pharmaceutical supply chain: pharmaceutical production, pharmaceutical distribution, and medical services; identifying systemically important institutions within the pharmaceutical supply chain; and analyzing changes in the tail risk contagion network under different risk events.

### 6.1 Conclusion

The research results show that: (1) From an overall perspective, the total connectedness of the pharmaceutical supply chain exhibits significant time-varying characteristics, especially during tail events, when the total connectedness is relatively high. At the module level, the production module acts as both the largest risk input and the largest risk output. At the cross-module level, risk contagion in the production module tends to be concentrated, and there are bidirectional risk spillovers between the production module and both the distribution and service modules. At the individual institutional level, the in-degree and out-degree of institutions do not depend on their market value. These findings collectively illuminate the structured nature of financial risk transmission within the pharmaceutical supply chain. Specifically, the distribution and propagation of risks across the network are not random but rather a systematic phenomenon shaped by three key dimensions: the position of entities within the industrial chain, the topological structure of the interconnected network, and the nature of external shocks. (2) Hengrui Medicine, Aier Eye Hospital, and Fosun Pharma are identified as systemically important institutions in the pharmaceutical supply chain’s tail risk spillover. Our study identifies systemically important institutions based on their centrality within the financial risk network, following the logic of being ‘too big to fail’ or ‘too connected to fail.’ This definition focuses on their potential impact on the financial stability of the entire industry. We acknowledge that this may not fully overlap with the definition of ‘critical institutions’ from a public health perspective, which emphasizes the provision of irreplaceable life-saving drugs or the guarantee of grassroots supply. However, understanding the vulnerabilities of these financial hubs is crucial. If such institutions encounter financial distress, the resulting pressure could transmit to numerous small and medium-sized enterprises upstream and downstream—including those critical from a public health standpoint—through channels such as supply chain credit, equity investments, and business expectations. This, in turn, could trigger or exacerbate broader medication supply risks. (3) The characteristics of the pharmaceutical supply chain risk network differ under different tail events. Financial crises increase the overall risk level of the supply chain, while public health events do not significantly raise the overall risk level. However, all tail risk events increase the frequency of risk transmission, especially the bidirectional risk spillover between the distribution and production modules, which requires heightened attention from regulatory authorities. This study focuses on the pharmaceutical industry in China, constructing a tail risk network to analyze the topological structure and dynamic spillover mechanisms of financial risk contagion within it. A financial crisis is inherently an endogenous shock to the financial system, characterized by liquidity drying up and asset price collapses. It directly and simultaneously impacts the valuations of all listed companies through credit channels, risk preference channels, and balance sheet channels. This is homogeneous and directly related to the financial tail risk measured by the TENET model. Therefore, a financial crisis inevitably triggers strong resonance within the financial risk network, manifesting as a sharp increase in total connectivity (TC). In contrast, when public health events occur, the pharmaceutical sector is widely regarded by capital markets as a “defensive asset” due to its inelastic demand characteristics. Investors anticipate that the government will make every effort to ensure the stability of the pharmaceutical industry, and certain sub-sectors (such as vaccines and testing) have clear positive prospects. This strong positive expectation and the influx of safe-haven funds support or even elevate the overall stock prices of listed pharmaceutical companies, thereby “hedging” the negative shocks in our tail risk model and resulting in no significant increase in overall risk levels (TC values). This explains why public health events did not significantly enhance overall connectivity, whereas financial crises did.

### 6.2 Policy recommendations

#### 6.2.1 A network-based framework for macro-prudential oversight

The significant fluctuations in Total Connectivity (TC), particularly its sharp rise during tail events, underscore the need for a dynamic monitoring system. We propose that financial and industry regulators integrate the TC index as a leading macro-prudential indicator. For instance, the establishment of a clear threshold—such as a TC value persistently exceeding two standard deviations above its historical trend—could serve as a trigger for enhanced sector-wide surveillance or mandatory stress testing. This would allow for pre-emptive action before localized distress propagates through the network.

#### 6.2.2 Targeted interventions for critical network nodes

Our identification of the production module as the primary risk hub—both absorbing and transmitting the most risk—calls for targeted, module-specific regulatory measures. Policymakers should consider implementing differentiated capital or liquidity buffers for systemically important entities within this module. Furthermore, to mitigate the observed intense bidirectional spillovers with distribution and service modules, regulatory guidelines could promote shorter inter-firm payment cycles or enhanced collateral requirements in transactions involving the production module. These measures would function as automatic stabilizers, dampening the amplification of shocks across the supply chain.

#### 6.2.3 Rethinking systemic importance: from size to connectivity

The finding that a firm’s risk influence (in-degree/out-degree) is not correlated with its market capitalization challenges the traditional “too-big-to-fail” focus. It necessitates a supplementary framework focused on “too-connected-to-fail.” Regulatory assessments should, therefore, incorporate metrics of network centrality (e.g., betweenness, eigenvector centrality) to identify firms that are critical due to their topological position. Such “high-connectivity” institutions, which may include medium-sized firms with essential niche roles, should be subject to closer monitoring of their supply chain dependencies and inter-firm exposures.

#### 6.2.4 A differentiated toolkit for heterogeneous shocks

Finally, the divergent impacts of financial crises and public health events demand a flexible, shock-specific response strategy. In a financial stress scenario (signaled by a rapidly rising TC), the policy priority should be swift, targeted liquidity provision to the core risk hubs (the production module and highly central firms) to prevent a credit freeze. Conversely, during a public health or operational crisis, where risk transmission frequency increases without a major TC spike, the focus must shift to ensuring operational resilience. This involves guaranteeing the physical and information flow integrity, for example, by creating prioritized “green lanes” for critical medical logistics and mandating real-time data sharing on inventory and capacity between key players to prevent coordination failures.

## 7 Limitations and future research directions

### 7.1 Sample selection limitations and improvements

We focus solely on listed companies and does not include a large number of non-listed small and medium-sized enterprises (SMEs). The structure of China’s pharmaceutical supply chain is highly fragmented, with SMEs playing critical roles in grassroots drug supply and regional markets. However, these SMEs generally exhibit weaker risk resilience and are more susceptible to external shocks. As a result, the measurement of tail risks in this study may somewhat underestimate the extent of risk exposure among SMEs and their contagion effects on the overall supply chain. Future research could integrate supply chain survey data, corporate credit information, or regional economic statistics to develop a more comprehensive risk monitoring framework, with particular emphasis on enhancing the risk characterization of non-listed entities.

### 7.2 Research timeliness and improvement

The sample data of this study ends in March 2023. While this covers the complete impact phase of the COVID-19 pandemic, it does not include the subsequent ‘post-pandemic’ recovery period or the more intensive industry regulatory changes that have occurred since the second half of 2023 (such as the nationwide anti-corruption campaign in the pharmaceutical sector). Future research will update the data to the latest available point to examine the dynamic reshaping effects of these new phases and factors on the pharmaceutical supply chain risk network.

### 7.3 Methodological limitations and improvements

The TENET model employed in our study is a powerful tool for analyzing tail risk networks; however, its application entails several inherent limitations. First, the model characterizes risk spillovers based on statistical conditional dependence, primarily revealing the channels and intensity of risk transmission rather than establishing strict causal mechanisms. Second, the smoothness assumption imposed on the link function g(⋅) may not fully capture abrupt or highly nonlinear features of risk transmission under extreme market conditions. Third, while the rolling-window setup captures dynamic changes, it may also smooth out some high-frequency, transient shock signals. Finally, the computational complexity of the model increases significantly with the number of institutions (N), which may constrain its applicability to very large-scale networks. Future research could integrate structured econometric models, such as incorporating exogenous instrumental variables, or employ high-frequency data causal discovery techniques. This would allow for the disentanglement of intrinsic causal chains while revealing the “risk transmission topology.” Additionally, exploring time-varying parameter settings and adaptive window algorithms could enhance the model’s ability to capture market regime shifts and transient shocks. Meanwhile, cutting-edge methods like graph neural networks offer new technological possibilities for handling ultra-large-scale risk networks.

## Data Availability

Publicly available datasets were analyzed in this study. This data can be found at: the macro state variables are selected from the Wind database (https://www.wind.com.cn/). The industry characteristic variables are derived from the quarterly balance sheets in the CSMAR database (https://www.gtarsc.com/).
